# YRS Survey

**DOI:** 10.5334/jbr-btr.1046

**Published:** 2016-02-16

**Authors:** Sofie Van Cauter, Maxime Bersou

**Affiliations:** 1 ZOL Genk, BE; 2 UCLouvain, BE

Residents have many questions concerning their training, education, and future, especially in a professional atmosphere that is considered difficult due to increasing financial and political restrictions in the health care system. To objectify this matter, the young radiologist section (YRS) created an online survey.

Two hundred and eleven Belgian residents and recently graduated radiologists were invited to answer this electronically based questionnaire within two years after the end of training. The survey was anonymous. Fifty-one questions had to be answered with a scaled response system from 1 to 5 corresponding to an opinion, ranging from “completely disagree” to “completely agree”. Questions were divided into five categories: perception of the quality of the residency, reporting system, attitude towards scientific research, career opportunities, and view on the Belgian Society of Radiology.

Results showed that residents and recently graduated radiologists are not undividedly satisfied about their training. There is a clear need for information and communication on the curriculum, goals, and expectations of the training. Young radiologists perceive there are differences in the quality of training across Belgian universities; therefore, a need for uniformisation in the residency is expressed. In that view, exchange between universities seems to be an opportunity, and a global interuniversity evaluation at the end of training should be considered.

Furthermore, the balance between workload on one hand and education and study on the other remains a juggling act. There is a clear need to create better conditions for scientific work to create awareness to novel evolutions and to prepare colleagues for an academic career. More than 90 per cent of participants responded positively to a subspecialisation in the last year(s) of training, which corresponds to the demands of the European training model in radiology.

In conclusion, this survey among residents and recently graduated radiologists showed several points of attention in the current Belgian radiology training. The YRS emphasizes that the radiology residency should be adapted to the quickly evolving landscape of today’s health care system.

## Competing Interests

The authors declare that they have no competing interests.

